# Juxtamembrane Shedding of *Plasmodium falciparum* AMA1 Is Sequence Independent and Essential, and Helps Evade Invasion-Inhibitory Antibodies

**DOI:** 10.1371/journal.ppat.1002448

**Published:** 2011-12-15

**Authors:** Anna Olivieri, Christine R. Collins, Fiona Hackett, Chrislaine Withers-Martinez, Joshua Marshall, Helen R. Flynn, J. Mark Skehel, Michael J. Blackman

**Affiliations:** 1 Division of Parasitology, MRC National Institute for Medical Research, Mill Hill, London, United Kingdom; 2 Protein Analysis and Proteomics Laboratory, Clare Hall Laboratories, Cancer Research UK London Research Institute, South Mimms, Hertfordshire, United Kingdom; University of Michigan, United States of America

## Abstract

The malarial life cycle involves repeated rounds of intraerythrocytic replication interspersed by host cell rupture which releases merozoites that rapidly invade fresh erythrocytes. Apical membrane antigen-1 (AMA1) is a merozoite protein that plays a critical role in invasion. Antibodies against AMA1 prevent invasion and can protect against malaria *in vivo*, so AMA1 is of interest as a malaria vaccine candidate. AMA1 is efficiently shed from the invading parasite surface, predominantly through juxtamembrane cleavage by a membrane-bound protease called SUB2, but also by limited intramembrane cleavage. We have investigated the structural requirements for shedding of *Plasmodium falciparum* AMA1 (PfAMA1), and the consequences of its inhibition. Mutagenesis of the intramembrane cleavage site by targeted homologous recombination abolished intramembrane cleavage with no effect on parasite viability *in vitro*. Examination of PfSUB2-mediated shedding of episomally-expressed PfAMA1 revealed that the position of cleavage is determined primarily by its distance from the parasite membrane. Certain mutations at the PfSUB2 cleavage site block shedding, and parasites expressing these non-cleavable forms of PfAMA1 on a background of expression of the wild type gene invade and replicate normally *in vitro*. The non-cleavable PfAMA1 is also functional in invasion. However – in contrast to the intramembrane cleavage site - mutations that block PfSUB2-mediated shedding could not be stably introduced into the genomic *pfama1* locus, indicating that some shedding of PfAMA1 by PfSUB2 is essential. Remarkably, parasites expressing shedding-resistant forms of PfAMA1 exhibit enhanced sensitivity to antibody-mediated inhibition of invasion. Drugs that inhibit PfSUB2 activity should block parasite replication and may also enhance the efficacy of vaccines based on AMA1 and other merozoite surface proteins.

## Author Summary

The malaria parasite invades red blood cells. During invasion several parasite proteins, including a vaccine candidate called PfAMA1, are clipped from the parasite surface. Most of this clipping is performed by an enzyme called PfSUB2, but some also occurs through intramembrane cleavage. The function of this shedding is unknown. We have examined the requirements for shedding of PfAMA1, and the effects of mutations that block shedding. Mutations that block intramembrane cleavage have no effect on the parasite. We then show that PfSUB2 does not recognise a specific amino acid sequence in PfAMA1, but rather cleaves it at a position determined primarily by its distance from the parasite membrane. Certain mutations at the PfSUB2 cleavage site prevent shedding, and parasites expressing non-cleavable PfAMA1 along with unmodified PfAMA1 grow normally. However, these mutations cannot be introduced into the parasite's genome, showing that some shedding by PfSUB2 is essential for parasite survival. Parasites expressing shedding-resistant mutants of PfAMA1 show enhanced sensitivity to invasion-inhibitory antibodies, suggesting that shedding of surface proteins during invasion helps the parasite to evade potentially protective antibodies. Drugs that inhibit PfSUB2 may prevent disease and enhance the efficacy of vaccines based on PfAMA1.

## Introduction

The phylum *Apicomplexa* contains several pathogens of major clinical and veterinary importance. These include the aetiological agents of coccidiosis in poultry, theileriosis and babesiosis in cattle, and toxoplasmosis, cryptosporidiosis and malaria in humans. In all cases, the causative agents are parasitic protozoa that share the feature of possessing several life-cycle stages that switch sequentially between replicative intracellular forms and transiently extracellular zoite stages equipped to seek out and invade suitable host cells. Invasion by apicomplexan zoites has been a subject of great attention for two major reasons: first, because it represents a step at which the parasite is exposed to host antibodies and other effector molecules capable of preventing invasion [1], [2]; and second, because it involves parasite-specific biochemical processes – including a number of protease-dependent modifications [3] and a specialised actinomyosin motor that drives motility and invasion (reviewed in [4]) - that are potential targets for the development of new antiparasite drugs.

The clinical manifestations of malaria stem from replication of asexual blood stages of *Plasmodium* spp. in circulating erythrocytes. In the case of the most dangerous malarial species, *Plasmodium falciparum*, the parasite develops intracellularly over the course of∼48 hours to produce a mature schizont containing around 16 daughter merozoites. The schizont eventually ruptures, releasing the merozoites which rapidly invade fresh erythrocytes, thus perpetuating the cycle. Erythrocyte invasion comprises several discrete steps [5]–[7], and is facilitated by the discharge of adhesive ligands and other proteins onto the parasite surface from specialised apical organelles called rhoptries and micronemes. Invasion is also accompanied by efficient proteolytic shedding of certain parasite surface and micronemal proteins. These include a micronemal type I integral membrane protein called AMA1 and an abundant glycosyl phosphatidylinositol-anchored merozoite surface protein called MSP1 (which forms a complex with two additional proteins called MSP6 and MSP7, collectively referred to as the MSP1/6/7 complex). Shedding of these proteins goes virtually to completion upon invasion [8]–[10]. Previous work from our group and others has demonstrated that shedding of *P. falciparum* AMA1 (PfAMA1) and the MSP1/6/7 complex, as well as another micronemal protein called PTRAMP, is all mediated by the same membrane-associated, calcium-dependent parasite subtilisin-like serine protease called PfSUB2, which is itself released from micronemes onto the merozoite surface during invasion [11]–[13]. Precise mapping of the single PfSUB2 cleavage sites in *P. falciparum* MSP1 and PfAMA1 has shown that the sequences that flank the scissile bond lack obvious similarity [12], [14], leading us to suggest that PfSUB2 may share characteristics with a number of vertebrate membrane-bound 'sheddases' that cleave relatively unstructured juxtamembrane regions of their protein substrates in a non sequence-dependent manner [15]–[20]. However, the requirements for substrate recognition by PfSUB2 have not been experimentally addressed.

In addition to PfSUB2-mediated shedding, under normal conditions of *in vitro* culture a small fraction of PfAMA1 is also shed by intramembrane cleavage [8], [12], likely mediated by either a parasite plasma membrane-localised rhomboid-like protease called PfROM4 [21] or a micronemal rhomboid called PfROM1 [22]. Given the relatively low levels of this intramembrane cleavage, we have previously postulated that it is physiologically unimportant in *P. falciparum*. Intriguingly, however, shedding of the *Toxoplasma gondii* AMA1 orthologue, TgAMA1, takes place exclusively through intramembrane cleavage [8], mediated predominantly through the action of the *Toxoplasma* orthologue of ROM4 [23], and recent findings suggests that this shedding plays a critical role in triggering parasite replication following invasion [24]. There is no known functional homologue of PfSUB2 in *Toxoplasma*, so the relative importance of PfSUB2-mediated and intramembrane shedding of PfAMA1 remains unclear.

Attempts to disrupt the single-copy *P. falciparum* PfSUB2 gene (*pfsub2*) or its orthologue in the rodent malaria species *P. berghei* have been unsuccessful, suggesting that SUB2 plays an indispensable role in parasite asexual blood-stages [11], [25]. Consistent with this, small molecules and monoclonal antibodies that bind structures close to the PfSUB2 cleavage site in MSP1 efficiently block shedding and also prevent invasion [26]–[29], suggesting that shedding of the MSP1/6/7 complex is important for invasion. An association between antibody-mediated inhibition of shedding and invasion has also been noted for antibodies against PfAMA1 [29]–[31], but other studies have suggested that certain antibodies to PfAMA1 block invasion primarily by preventing its interactions with a set of rhoptry neck-derived parasite partner proteins (RON proteins) that are thought to associate with PfAMA1 at the moving junction during invasion [32]–[36]. There is to date no firm evidence that shedding of PfAMA1 is important for parasite viability.

Like SUB2, both MSP1 and AMA1 are also indispensable in the asexual blood stage life cycle, indicating that they play critical roles [37]–[40]. Whilst MSP1 has orthologues only in other *Plasmodium* species, AMA1 is conserved in most other apicomplexan genomes examined [41]–[43]. In addition to its putative role in the transition to replicative growth referred to above, AMA1 has also been implicated in signalling events during invasion [44], [45]. Both AMA1 and MSP1 are widely considered potential malaria vaccine candidates, since they can induce antibody responses that block invasion *in vitro* and protect against blood-stage malaria *in vivo* (see [46] and [47] for recent reviews). Many of the *in vitro* studies have found that substantial concentrations of specific antibody (usually>0.1 mg ml^−1^) are required to obtain a significant effect on invasion (e.g. [9], [48]–[50]. Perhaps in part as a result of this, clinical trials of vaccines based on these two proteins have thus far proved disappointing [51]–[54]. Shedding of apicomplexan zoite surface proteins during invasion is frequently observed across the phylum (reviewed in [55]), provoking speculation regarding its general role. One proposal - that shedding may be required to disengage adhesive interactions between parasite ligands and host cell receptors - has received substantial experimental support [21], [23], [56]. In addition, shedding may allow evasion of invasion-inhibitory antibodies during invasion, as the shed molecules could bind and deplete serum antibodies in the microenvironment of the parasite-host cell interaction, as well as minimizing accumulation of immune complexes on the parasite surface [57]. Though intuitively attractive, there is no evidence supporting the latter hypothesis.

Here we have investigated the structural requirements for shedding of PfAMA1, and the consequences of its inhibition. We first show that intramembrane cleavage of PfAMA1 can be reduced to virtually undetectable levels by mutagenesis, with no discernible phenotypic consequences. We then show that the position of cleavage by PfSUB2 is determined primarily not by the sequence at that site but by its distance from the transmembrane domain (TMD). Despite this, certain radical mutations at the PfSUB2 cleavage site can block shedding. Parasites expressing these non-cleavable forms of PfAMA1 on a background of expression of the wild type protein invade and replicate normally *in vitro*. However, the same mutations that block PfSUB2-mediated shedding could not be stably introduced into the parasite genome, suggesting that some shedding of PfAMA1 by PfSUB2 is essential. Furthermore, parasites expressing shedding-resistant mutants of PfAMA1 are markedly more sensitive to antibody-mediated inhibition of invasion than those expressing wild-type forms of PfAMA1, suggesting that shedding of surface proteins during invasion by apicomplexan zoites may also serve as a mechanism to evade potentially protective invasion-inhibitory serum antibodies.

## Results

### Mutation of the intramembrane cleavage site in the PfAMA1 TMD abolishes cleavage with no effect on parasite viability

**Figure 1:**
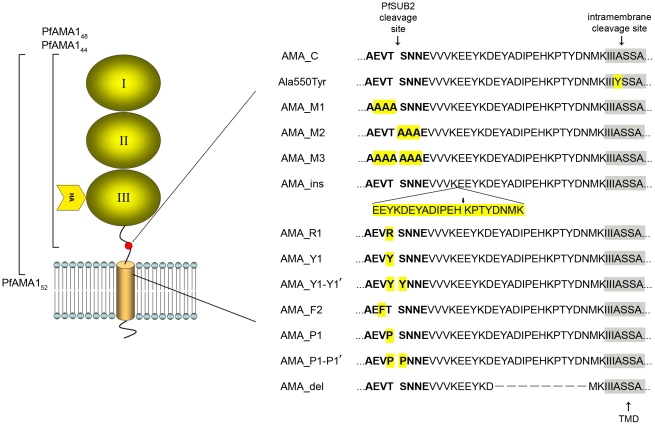
Mutants and antibodies used in this study. The overall architecture of PfAMA1/DIII-HA is shown schematically (left; not to scale), including the three domains making up the PfAMA1 ectodomain (numbered), the position of the HA epitope tag within the membrane-proximal domain III, the transmembrane domain (TMD; shown as a cylinder), and short cytoplasmic domain. The position of PfSUB2 cleavage, which lies 29 residues from the TMD between Thr517 and Ser518 [12], is indicated with a red dot. The nomenclature used for shed fragments of the ectodomain resulting from juxtamembrane or intramembrane cleavage are indicated on the left. Note that PfAMA1_44_ is derived from PfAMA1_48_ as a result of an internal 'nick' within domain III; both are a result of shedding at the PfSUB2 site, whereas PfAMA1_52_ arises from intramembrane shedding [12]. On the right are shown the names of the parental construct (called AMA_C) for episomal expression of unmodified PfAMA1/DIII-HA, plus the various mutant constructs, together with their corresponding sequence between the PfSUB2 cleavage site (arrowed, with flanking residues in bold) and the TMD (shaded), which contains the intramembrane cleavage site (arrowed). Substitutions and insertions made are highlighted in yellow, whilst residues removed in mutant AMA_del are indicated as dashes. The arrow against the AMA_ins sequence shows the predicted position of PfSUB2 cleavage in this sequence if it is determined primarily by its distance from the TMD. The Ala550Tyr mutation was not expressed episomally, but introduced into the genomic *pfama1* gene as described in the text. The rat anti-PfAMA1 monoclonal antibody 4G2 used in this work recognizes an epitope formed by domains I and II of PfAMA1 [32], [63], whilst the rabbit and mouse polyclonal anti-PfAMA1 antibodies used were raised against a recombinant, correctly-folded form of the entire ectodomain, expressed in *Pichia pastoris* [32], [64].

**Figure 2:**
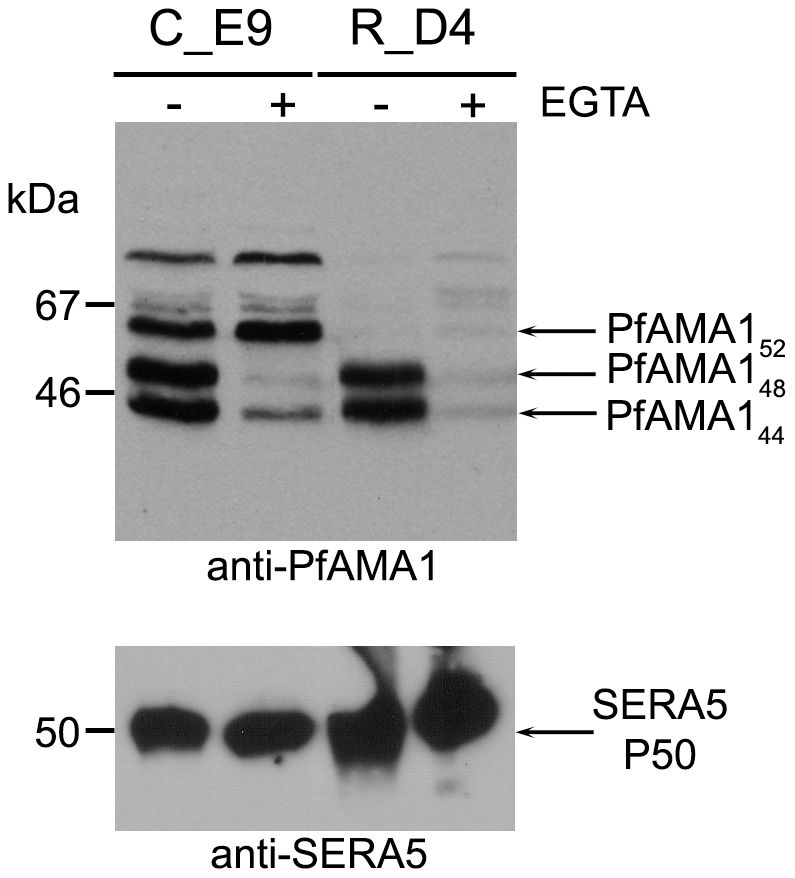
Mutation of the intramembrane cleavage site in PfAMA1 inhibits shedding by intramembrane processing. Western blot analysis of culture supernatants from transgenic *P. falciparum* clones 3D7_AMA_C_E9 (control) and 3D7_AMA_R_D4 (Ala550Tyr), following schizont rupture in the presence or absence of 10 mM EGTA. Samples were electrophoresed under reducing conditions and probed with anti-PfAMA1 polyclonal serum R5 (top) or monoclonal antibody 24C6.1F1 specific for the parasitophorous vacuole protein SERA5, which is released in the form of the P50 fragment (bottom – loading control). For both clones, shedding of PfAMA1 by PfSUB2 gives rise to the expected products of PfAMA1_48_ plus PfAMA1_44_ ([12] and Figure 1). In addition, PfAMA1 is shed from the surface of 3D7_AMA_C_E9 parasites via intra-membrane cleavage, giving rise to PfAMA1_52_. This species is absent from 3D7_AMA_R_D4 culture supernatants, confirming that the introduced Ala550Tyr substitution blocks intramembrane processing of PfAMA1. Because PfSUB2-mediated shedding is calcium-dependent, virtually all PfAMA1 shedding is ablated from the 3D7_AMA_R_D4 parasites in the presence of EGTA.

Precise mapping by us and others of the intramembrane cleavage sites in PfAMA1 and TgAMA1 [8], as well as in the *P. falciparum* microneme protein EBA-175 [21] and the other *Toxoplasma* microneme proteins MIC2 [58] and MIC6 [59], has shown in all cases that cleavage takes place on the C-terminal side of an Ala residue (the P1 position in Schechter and Berger terminology [60]). No detailed information is available about the specific substrate requirements of PfROM4 or PfROM1, the rhomboid-like enzymes potentially responsible for intramembrane cleavage of PfAMA1. However, studies of several other rhomboids from evolutionarily diverse organisms have indicated that only residues with a small side-chain, such as Ala, Cys, Ser and Gly, can be accommodated at P1, and that cleavage can often be prevented by substitution of the P1 residue with bulky residues such as Tyr or Phe [61], [62]. To investigate the importance of intramembrane shedding of PfAMA1, we therefore produced a construct designed to modify the endogenous *pfama1* gene by targeted homologous recombination, replacing the P1 Ala550 with a Tyr residue (Figure 1). Parasites transfected either with this construct (called intAMA_R-TKmod) or with control construct intAMA_C-TKmod (which is identical to intAMA_R-TKmod aside from the Ala550Tyr substitution, and is thus designed to simply reconstitute the authentic PfAMA1 TMD sequence) were maintained in the presence of WR99210. After 2 cycles of drug selection and ganciclovir treatment to enrich for integration events, both parasite lines were cloned by limiting dilution. Southern blot analysis of the obtained clones (Figure S1 in Supporting Information Text S1) showed that integration of both constructs had occurred in the expected manner, resulting in transgenic parasites that should express PfAMA1 with either the Ala550Tyr substitution or an unmodified TMD. Growth assays showed no discernible growth defect in any of the clones, and immunofluorescence analysis (IFA) and Western blot with antibodies to PfAMA1 indicated no alteration in the sub-cellular pattern or levels of PfAMA1 expression in schizonts (not shown). To assess the effect of the mutation on shedding of PfAMA1, mature schizonts enriched from synchronised cultures of clones 3D7_AMA_C_E9 (control) and 3D7_AMA_R_D4 (Ala550Tyr) were allowed to undergo rupture in either normal medium, or medium supplemented with the calcium chelator EGTA. This compound has no effect on schizont rupture under the conditions used, but efficiently inhibits both invasion and PfSUB2-mediated shedding (which is calcium-dependent) whilst simultaneously enhancing shedding by intramembrane cleavage, presumably due to accumulation of PfAMA1 on the surface of released merozoites [8], [12]. As shown in Figure 2, the usual products of PfSUB2 cleavage, PfAMA1_44_ and PfAMA1_48_, were equally abundant in normal culture supernatants from both clones. However, the 3D7_AMA_R_D4 supernatants were clearly deficient in the PfAMA1_52_ fragment which derives from intramembrane cleavage. Furthermore, whereas in the case of control clone 3D7_AMA_C_E9 the addition of EGTA resulted in a relative increase in levels of shed PfAMA1_52_, as expected, the same treatment severely ablated all PfAMA1 shedding in the mutant 3D7_AMA_R_D4 clone. Titration of the culture supernatants by Western blot showed that the Ala550Tyr substitution reduced production of the PfAMA1_52_ fragment by a factor of at least 8-fold (Figure S2 in Text S1). These results convincingly demonstrate that the Ala550Tyr substitution in the PfAMA1 TMD efficiently blocks shedding of PfAMA1 by intramembrane cleavage. The fact that the mutant clones show no growth phenotype *in vitro* strongly suggests that intramembrane shedding of PfAMA1 does not play a critical role in the asexual blood-stage cycle of *P. falciparum*, or at least that only very low levels of intramembrane cleavage (close to or below those detectable by Western blot) are sufficient for normal parasite growth.

### Non-sequence-dependent cleavage of PfAMA1 by PfSUB2

**Figure 3:**
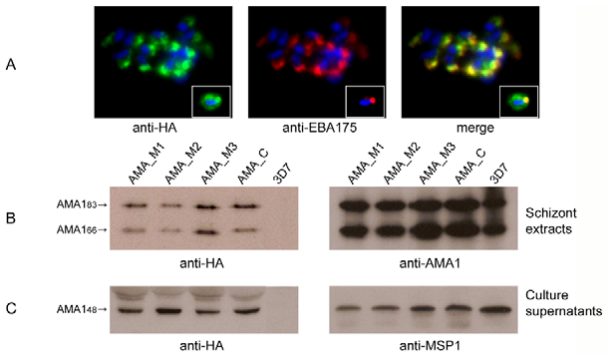
Alanine mutagenesis of the PfSUB2 cleavage site does not affect expression, localisation or shedding of PfAMA1. (A) IFA of drug-selected 3D7 parasites harbouring the AMA_M3 construct. The transgenic PfAMA1/DIII-HA product detected by anti-HA mAb 3F10 (green) co-localises with the microneme protein EBA-175 (red) in mature segmented schizonts. The inset shows similar staining of a free merozoite, where some translocation of PfAMA1/DIII-HA onto the merozoite surface is detectable as is normal for endogenous PfAMA1. Identical IFA patterns were observed in parasites harbouring mutant constructs AMA_M1 and AMA_M2 (not shown). (B) Western blot of parental 3D7 and transgenic parasite lines harbouring construct AMA_C and mutants thereof. All show the characteristic doublet corresponding to PfAMA1_83_ (the full-length protein minus signal peptide) and PfAMA1_66_ (PfAMA1_83_ minus its prosequence) [12], [66]. The transgene products are detected by anti-HA mAb 3F10 in all lines except the parental 3D7 clone. (C) Western blot of culture supernatants collected from the above lines. The shed PfAMA1_48_ fragment derived from the transgenes is detected with anti-HA mAb 3F10, whilst a mAb (X509) reactive with a shed fragment of MSP1 (MSP1_33_) serves as a loading control. Note that the single HA tag in PfAMA1/DIII-HA spans the site of the internal 'nick' in domain III that normally produces PfAMA1_44_, preventing the usual partial cleavage at that site [32]; PfAMA1_44_ is therefore never observed when probing culture supernatants with the anti-HA antibodies.

Having shown to our satisfaction that normal levels of intramembrane shedding of PfAMA1 are not essential in asexual blood-stages, we turned our attention to the importance and mechanism of juxtamembrane shedding. In previous work [32], [63], [64] we have described episomal expression of a synthetic recodonised FVO *pfama1* transgene in *P. falciparum*. Expressed under the control of the *pfama1* promoter, the transgene product (called PfAMA1/DIII-HA; Figure 1) is correctly trafficked to micronemes and eventually to the merozoite surface, where it is correctly cleaved and shed at erythrocyte invasion. The presence of a single hemagglutinin (HA) epitope tag in the domain III loop of the transgene product allows it to be discriminated from endogenous PfAMA1, which continues to be expressed in the transgenic lines. For the present study, we decided to use this same episomal expression system to examine the sequence requirements for cleavage by PfSUB2. To do this, we expressed a set of mutant forms of PfAMA1/DIII-HA in *P. falciparum* and examined their expression and proteolytic shedding. Most proteases recognise specific amino acid residues immediately flanking the scissile bond, so in initial experiments we examined the importance of these residues in processing by PfSUB2. Constructs for episomal expression of three mutants of PfAMA1/DIII-HA were first generated, incorporating Ala substitutions of all three residues just upstream of the cleavage site (positions P3–P1; mutant AMA_M1), or the three residues downstream of the processing site (P1′–P3′; mutant AMA_M2), or all six residues (P3–P3′; mutant AMA_M3) (Figure 1). As shown in Figure 3 panels A and B, examination by IFA and Western blot using an HA-specific antibody showed that all three mutant transgene products were expressed in transfected parasites at levels equivalent to those of the parental construct (AMA_C), and exhibited a similar pattern of sub-cellular localisation and intracellular proteolytic prosequence removal as is usually observed for PfAMA1 [65], [66]. Most notably, examination of culture supernatants harvested following schizont rupture and merozoite release revealed, in all cases, the presence of the PfAMA1_48_ species diagnostic of correct ectodomain shedding by PfSUB2 (Figure 3C). Under carefully standardised culture conditions there were no reproducible differences in the degree of shedding or the apparent molecular mass of the shed PfAMA1_48_ protein on SDS-PAGE. These results strongly suggest that cleavage of PfAMA1 by PfSUB2 at the bond between residues 517–518 can occur efficiently irrespective of the precise amino acid sequence immediately flanking the scissile bond.

### The PfSUB2 cleavage site in PfAMA1 is primarily determined by its distance from the transmembrane domain

**Figure 4:**
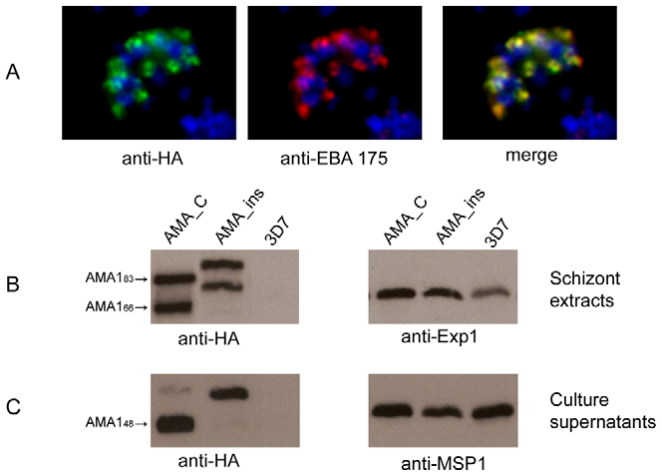
The site of PfSUB2 cleavage in PfAMA1 depends primarily upon its distance from the TMD. (A) IFA of drug-selected 3D7 parasites harbouring the AMA_ins construct. The transgene product detected by anti-HA mAb 3F10 (green) co-localises correctly with the microneme protein EBA-175 (red) in mature segmented schizonts. (B) Western blot of parental 3D7 and transgenic parasite lines harbouring constructs AMA_C and AMA_ins. The transgene-derived PfAMA1_83_ and PfAMA1_66_ products are detected by anti-HA mAb 3F10. Note that both species migrate more slowly in the case of AMA_ins, consistent with the 21-residue insertion in this construct. An antibody reactive with the parasite membrane protein Exp1 is used as a loading control. (C) Western blot of culture supernatants collected from the above lines. The shed transgenic PfAMA1_48_ fragment (detected with anti-HA mAb 3F10) is clearly of higher molecular mass in the case of AMA_ins than in the case of the 'wild-type' AMA_C construct. A mAb (X509) reactive with a shed fragment of MSP1 (MSP1_33_) serves as a loading control.

A number of reports examining proteolytic cleavage of membrane-bound proteins by membrane-associated sheddases have shown that the site selected for cleavage, rather than being dependent on its precise flanking sequence, is instead determined by its distance from the membrane and its location within a relatively unstructured juxtamembrane segment [15], [16]–[20]. In the light of our above results, we decided to exploit further mutants of PfAMA1/DIII-HA to investigate whether a similar rule holds true for PfSUB2 cleavage of PfAMA1. Mutant construct AMA_ins (Figure 1) was designed to contain a perfect duplication of 21 residues (Glu526 – Lys546 inclusive) that lie within the residual 'stub' that is normally left on the merozoite surface after PfSUB2-mediated shedding of the bulk of the PfAMA1 ectodomain. As a result, in AMA_ins the normal Thr517-Ser518 PfSUB2 cleavage site within the motif AEVT↓SNNE is shifted substantially further away from the predicted TMD than its position in the wild-type protein. We hypothesised that, should this mutant remain sensitive to PfSUB2, cleavage would occur in one of two manners dependent upon the mechanism of substrate recognition. If cleavage was strictly sequence-specific, cleavage would occur as usual at the Thr517–Ser518 bond, resulting in the release of the normal PfAMA1_48_ product. If, on the other hand, the site of cleavage was dependent on its distance from the membrane, cleavage would now take place at a new site, probably at or around the position indicated in Figure 1, since this would now lie 29 residues from the TMD; importantly, the latter scenario would result in the shedding of a∼2.5 kDa larger form of PfAMA1_48_. As shown in Figure 4A and 4B, episomal expression of AMA_ins in transfected *P. falciparum* resulted in the usual pattern of microneme localisation, and the expression as expected of larger forms of intracellular anti-HA-reactive PfAMA1, likely due to the presence of the 21 residue (∼2.6 kDa) insertion in AMA_ins. Examination of culture supernatants from this line (Figure 4C), showed that the shed form of AMA_ins migrated on SDS-PAGE substantially more slowly than the AMA_C-derived 'wild-type' PfAMA1_48_ species. These results are consistent with the notion that PfSUB2-mediated shedding of PfAMA1 is not primarily dependent on the flanking sequence but takes place at a constant distance from the TMD, irrespective of the amino acid sequence.

To seek a more precise estimate of the position of PfSUB2-mediated cleavage in the AMA_ins transgene product, we decided to isolate the shed product from culture supernatants for more detailed structural examination. Attempts to affinity-purify it using anti-HA antibodies failed, possibly due to poor solvent accessibility of the domain III-located single HA epitope tag in the folded protein. However, using a previously described method [66], we successfully affinity-purified the shed protein using the anti-PfAMA1 monoclonal antibody (mAb) 4G2. This mAb recognises both the transgene product and endogenous PfAMA1, so the purified preparations contained both forms of the protein; however, staining and Western blot analysis with anti-HA antibodies clearly discriminated the AMA_ins product, which migrates slightly more slowly than the endogenous PfAMA1_52_ species (Figure S3 in Text S1). Examination by LC/MS/MS of in-gel tryptic digests of the AMA_ins fragment unambiguously identified peptides spanning most of the predicted ectodomain downstream of the known position of prosequence cleavage at Ile97 [66] (Figure S4 in Text S1). Importantly, the most C-terminal tryptic peptide identified, AEVTSNNEVVVK (predicted monoisotopic *m/z* 1,288.67), has never been observed in digests of wild-type PfAMA1_48_ [12], confirming that cleavage of AMA_ins takes place at a site downstream of the normal AEVT↓SNNE site (see Figure 1). We were unable to identify any peptide species resembling DEYADIPEH, the expected C-terminal product of tryptic digestion if cleavage of AMA_ins takes place as predicted in Figure 1. Nonetheless, the presence of the AEVTSNNEVVVK peptide, and absence of additional tryptic peptides C-terminal to that sequence supports our prediction that cleavage of AMA_ins occurs at or around the IPEH↓KPTY motif. This in turn supports the notion that PfSUB2-mediated shedding of PfAMA1 is primarily dependent on its distance from the TMD and is not constrained by primary sequence determinants flanking the normal AEVT↓SNNE site.

Expression of transgenes from episomal constructs in *P. falciparum* is complicated by the fact that, even under continuous selective drug pressure, episomes generally segregate poorly in the parasite [67]. The result of this is that, at any one time, only a fraction of the parasites in a drug-selected population express the transgene of interest. Estimation of this fraction is possible using antibodies specific for the transgene. IFA analysis of the various lines described above using anti-HA and anti-PfAMA1 antibodies showed reproducibly that ∼30% of schizonts expressing PfAMA1 also exhibited expression of the transgenic PfAMA1/DIII-HA (data not shown). On the other hand, accurate quantification of relative abundance of the transgene product and endogenous gene product in these parasites can be difficult, especially when (as in the case of most of the mutants described above) the transgene product migrates on SDS-PAGE similarly to the endogenous gene product. Expression of the AMA_ins mutant, however, allowed us to take advantage of its distinctly slower migration characteristics to estimate its expression levels relative to that of endogenous PfAMA1. As shown in Figure S5 in Text S1, titration by Western blot using polyclonal antibodies to PfAMA1 revealed that ∼15% of the total PfAMA1 expressed by the AMA_ins line was in the form of the larger AMA_ins products. This is also in approximate concordance with the stained SDS PAGE analysis of the affinity-purified protein shown in Figure S3 in Text S1. Taken together with the IFA data this allowed us to estimate that, in those individual parasites harbouring the episome, ∼50% (15/30%) of the total PfAMA1 expressed was derived from the transgene. Since all PfAMA1/DIII-HA-expressing constructs used in this study shared a common plasmid backbone and regulatory sequence, we considered it reasonable to assume that this value holds for all mutants examined. This was important for the interpretation of subsequent experiments, as described below.

### Radical mutagenesis of the PfSUB2 cleavage site blocks shedding of PfAMA1

**Figure 5:**
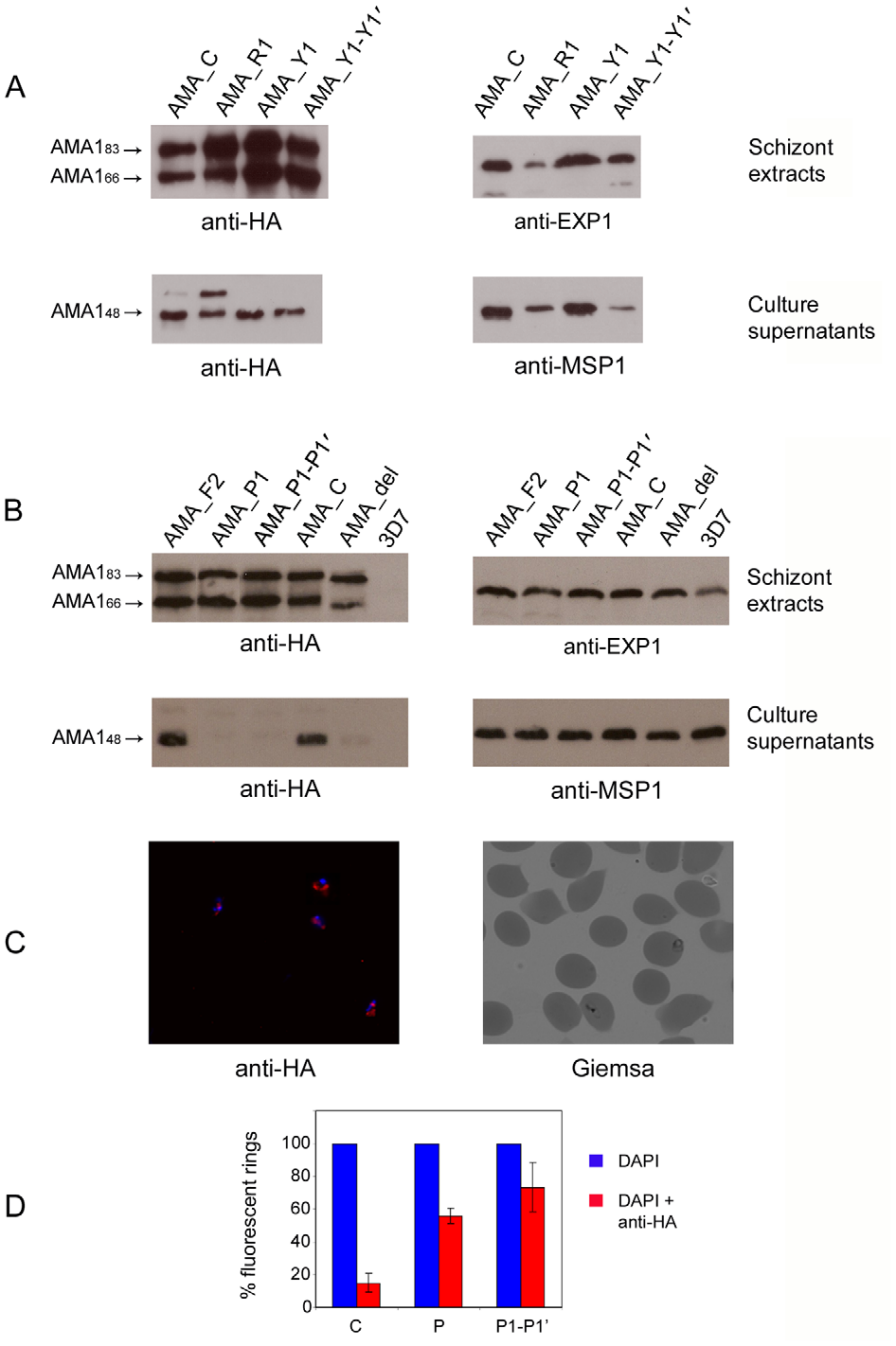
Radical mutagenesis of the cleavage site prevents PfSUB2-mediated shedding of PfAMA1. (A) and (B) Western blot of schizont extracts and culture supernatants of parental 3D7 and transgenic parasite lines harbouring constructs AMA_C (included in both panels A and B as a control), AMA_R1, AMA_Y1, AMA_Y1-Y1', AMA_F2, AMA_P1, AMA_P1-P1′, and AMA_del. The transgene-derived PfAMA1_83_ and PfAMA1_66_ products in schizont extracts are detected by anti-HA mAb 3F10. An antibody reactive with the parasitophorous vacuole membrane protein Exp1 serves as a loading control. Culture supernatants collected from the above lines were also probed with the anti-HA mAb 3F10. An antibody (mAb X509) reactive with a shed fragment of MSP1 (MSP1_33_) serves as a loading control. Note that the shed transgenic PfAMA1_48_ fragment is virtually absent from supernatants of the AMA_P1, AMA_P1-P1′ and AMA_del mutants. (C) IFA of newly-invaded ring stages of the parasite line harbouring construct AMA_P1. Note that, in contrast to the AMA_C line (not shown), many of the rings detectable with the DNA-reactive dye 4,6-diamidino-2-phenylindole (DAPI; blue) demonstrated strong reactivity with the anti-HA mAb 3F10 (red). The Giemsa-stained field is from a different, stained thin film from the same ring preparation. (D) Quantification of the proportion of anti-HA reactive rings in the AMA_P1, AMA_P1-P1′ and AMA_C lines. The error bars represent standard deviation (SD) values calculated from three different counts of the same experiment.

As described in the Introduction, it has been suggested that efficient proteolytic shedding of PfAMA1 from the merozoite surface is a prerequisite for successful erythrocyte invasion. In view of our findings that straightforward Ala substitution of residues immediately flanking the PfSUB2 cleavage site in PfAMA1 has no effect on PfSUB2-mediated shedding, we decided to produce a further series of mutant PfAMA1/DIII-HA expression constructs designed to identify more radical substitutions around this site that might prevent shedding. To assess the sensitivity of cleavage to charged or large residues at the P1 and P1′ positions, constructs AMA_R1, AMA_Y1 and AMA_Y1-Y1′ were first designed, in which either the P1 position only was replaced with Arg or Tyr, or both P1 and P1′ positions were replaced with Tyr, respectively. In addition, mutant AMA_F2 was produced, in which the P2 Val residue was substituted with a Phe residue. Many proteases are unable to cleave adjacent to Pro residues in a peptide chain, due to the cyclic nature and conformational rigidity of the associated imido bond; indeed, two adjacent Pro residues provide a high degree of resistance to most proteases [68]. Accordingly, mutant constructs AMA_P1 and AMA_P1-P1′ were also produced, respectively encoding Pro residues in positions P1 only, or in both P1 and P1′ relative to the PfSUB2 cleavage site (Figure 1). The final mutant produced was AMA_del, in which 14 residues (Glu531–Asn544 inclusive) that lie between the PfSUB2 cleavage site and the TMD were deleted (Figure 1). This was predicted to have the effect of bringing the globular domains I–III of PfAMA1/DIII-HA closer to the membrane, rendering the cleavage site less accessible to PfSUB2. It was also expected to result in a gene product ∼1.7 kDa smaller than the non-mutated PfAMA1/DIII-HA expressed from construct AMA_C. Transfection of these constructs into *P. falciparum* resulted in expression of all 7 transgene products at levels approximately equivalent to those of the parental construct AMA_C, with the same pattern of sub-cellular localisation (not shown) and intracellular proteolytic prosequence cleavage (Figure 5A–B). Examination of culture supernatants from the parasite lines showed that shedding of the AMA_Y1, AMA_Y1-Y1′ and AMA_F2 transgene products was as efficient as that of the control protein, supporting the conclusions of the alanine mutagenesis studies that PfSUB2 shedding is not dependent on the precise sequence at the cleavage site. Shedding of the AMA_R1 mutant was also reproducibly efficient, although in this case enhanced levels of the intramembrane cleavage product PfAMA1_52_ were also evident (Figure 5A). However, shedding of the other mutant transgenes was substantially less efficient, with decreased shedding of the AMA_del mutant and virtually complete absence of shedding in the case of the AMA_P1 and AMA_P1–P1′ mutants (Figure 5B). Further analysis of these latter two lines by IFA showed a substantially increased proportion of anti-HA reactive newly-invaded rings relative to the AMA_C control, consistent with a severe defect in shedding of the PfAMA1 ectodomain at invasion (Figure 5C and 5D). The anti-HA signal in these rings partially co-localised with that of a monoclonal antibody against MSP1_19_, the GPI-anchored MSP1-derived fragment that remains on the merozoite surface following PfSUB2-mediated cleavage of this protein at invasion (see Figure S6 in Text S1). This shows that at least some of the transgenic PfAMA1 carried into the rings was at the parasite plasma membrane and not still resident in micronemes. Despite this, there were no significant differences in growth rates between the control transgenic line and any of the mutant lines over>20 growth cycles (data not shown). Collectively, these results demonstrate that certain major modifications of the PfSUB2 cleavage site within PfAMA1 can effectively modify or block its shedding by the protease, but that – under conditions of episomal PfAMA1 expression on a background of expression of the genomic wild-type gene – this is not detectably deleterious to parasite growth *in vitro*.

### Some PfSUB2-mediated shedding of PfAMA1 is essential in asexual blood-stages

**Figure 6:**
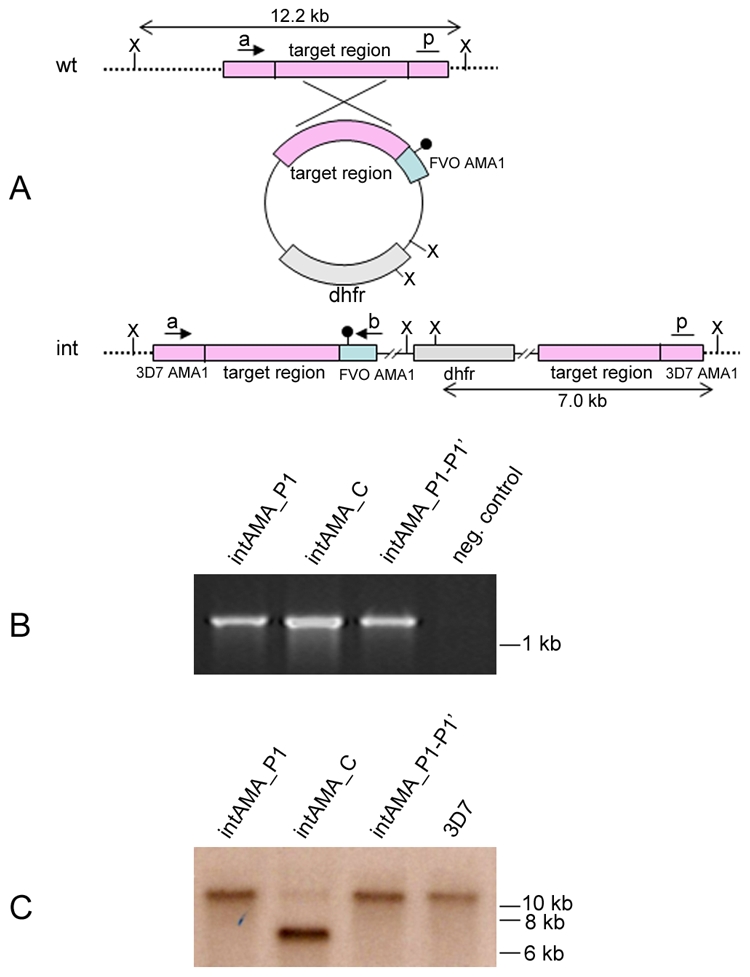
PfSUB2-mediated shedding of endogenous PfAMA1 is essential for asexual blood-stage parasite viability. (A) Schematic representing integration construct intAMA_C (and mutant forms intAMA_P1 and intAMA_P1-P1′) designed to modify the *pfama1* gene by single-crossover homologous recombination. The wild-type *pfama1* locus (wt, top) and the modified locus arising from the expected integration even (int) are also shown. *Xba*I sites (X) used for Southern blot analysis, and the relative positions of oligonucleotide primers used for diagnostic PCR are also indicated (a, primer verAMAint_F; b, primer verAMAint_R – see Table S1 in Text S1). Authentic 3D7 *pfama1* coding sequence is shown in pink, whilst recodonised sequence encoding the FVO *pfama1* coding sequence is in light blue. The position of the PfSUB2 cleavage site is indicated (black lollypop symbol). p, position of probe used for Southern blot analysis. (B) PCR analysis of genomic DNA from wild-type parent 3D7 and transgenic parasite lines harbouring constructs intAMA_C, intAMA_P1 and intAMA_P1-P1' using primers verAMAint_F and verAMAint_R, schematically represented in panel A. (C) Southern blot analysis of *Xba*I-digested genomic DNA from wild-type 3D7 and transgenic lines harbouring constructs intAMA_C, intAMA_P1 and intAMA_P1-P1'. The expected sizes of fragments hybridizing with the probe were 12.2 kb for the unmodified wild type locus and 7.0 kb in the case of the expected integration event.

In all the transgenic lines described above, expression of PfAMA1/DIII-HA or mutants thereof takes place on a background of expression of the authentic wild-type *pfama1* gene from its genomic locus. Encouraged by our identification of mutations capable of efficiently blocking shedding of the PfAMA1/DIII-HA transgene products, we sought to determine whether these same mutations could be introduced into the genomic *pfama1* gene, as previously achieved with the Ala550Tyr that blocks intramembrane cleavage. This would be predicted to block *all* PfSUB2-mediated shedding of endogenous PfAMA1, enabling us to determine whether any shedding of the protein is required for asexual blood-stage parasite viability. To do this, three new constructs were produced, each designed to integrate into the *pfama1* locus by single-crossover homologous recombination. Whereas integration of construct intAMA_C would effectively reconstitute the sequence encoding the wild-type gene product (thus acting as a control), integration of constructs intAMA_P1 and intAMA_P1-P1′ was predicted to introduce Pro substitutions of residues at either the P1 position or both the P1 and P1′ positions respectively relative to the PfSUB2 cleavage site (Figure 6A).

The three constructs were independently introduced into 3D7 parasites by transfection, and integrants selected for using a standard drug cycling protocol. As shown in Figure 6B, in 2 independent experiments, PCR analysis of drug-resistant parasite populations after 2 cycles of drug selection clearly detected the predicted integration events in all cases, suggesting that parasites in which integration had occurred were at least transiently viable. However, examination of drug-selected lines by Southern blot after 3 drug cycles (Figure 6C) showed integration only in the case of construct intAMA_C, strongly suggesting that introduction of either the P1 or P1-P1′ Pro substitutions was deleterious to parasite replication. Identical results were obtained using another set of constructs, called intAMA_C_TK, intAMA_P1_TK and intAMA_P1-P1'_TK, based on a pHHT-TK vector backbone (not shown). Our findings suggest that complete blockade of PfSUB2-mediated shedding of PfAMA1 impacts on parasite viability and so cannot be tolerated by the parasite.

### Shedding-resistant forms of PfAMA1 are functional in invasion

**Figure 7:**
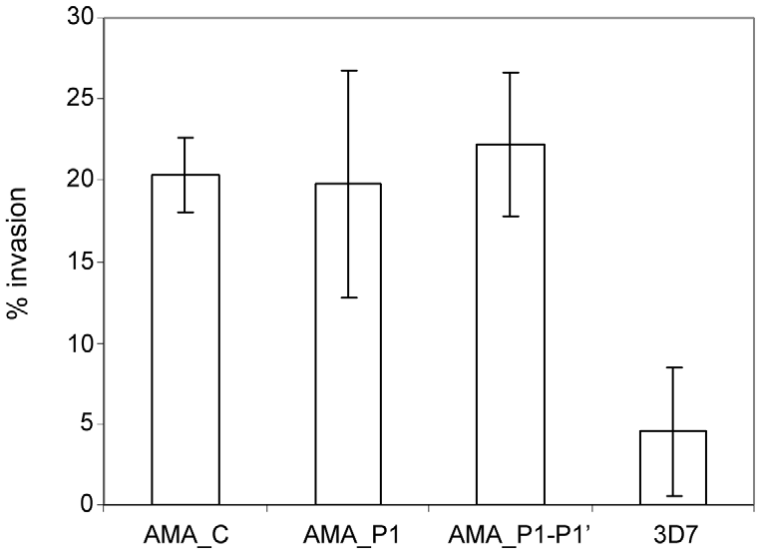
Functional complementation by episomally-expressed PfAMA1 is unaffected by mutations that block PfSUB2-mediated shedding. Histogram showing the effects of peptide R1 (100 µg ml^−1^ final concentration) on erythrocyte invasion by wild type 3D7 parasites or transgenic lines harbouring constructs AMA_C, AMA_P1 or AMA_P1-P1′. Normalised relative invasion efficiencies (expressed as % of controls) were calculated by dividing the parasitaemia values of the R1-treated cultures with those of parallel cultures allowed to undergo invasion in the absence of R1. Error bars indicate standard deviation values, calculated from 5 independent experiments.

Having failed to obtain viable parasites in which the endogenous *pfama1* gene was modified so as to block PfAMA1 shedding, we turned our attention back to the transgenic parasite lines episomally expressing shedding-resistant mutants of PfAMA1. PfAMA1 is a remarkably polymorphic molecule, with documented naturally-occurring amino acid substitutions in at least 52 positions within the ectodomain [69]. Previous work from Foley and colleagues has identified a 20-residue peptide called R1 which binds to PfAMA1 in an allele-specific manner [70], [71]. R1 potently inhibits erythrocyte invasion by those parasite strains that express forms of PfAMA1 bound by the peptide, probably by preventing interactions between PfAMA1 and its rhoptry neck-derived RON partner proteins at invasion [72]. In two previous studies of PfAMA1 function this strain-specific invasion-inhibitory activity of R1 was elegantly exploited to evaluate functional complementation by a series of episomally-expressed PfAMA1 mutants containing modifications within the cytoplasmic domain [44], [45]. We decided to take a similar approach to study the functional competence of our episomally-expressed, shedding-resistant PfAMA1 mutants. These are all based on the FVO PfAMA1, which is not bound by R1, whereas the parental 3D7 parasite clone used throughout this work is fully sensitive to R1 [70]. Lines harbouring AMA_C, AMA_P1 and AMA_P1-P1′ were synchronised in parallel with the parental 3D7 clone, then allowed to undergo erythrocyte invasion in the presence or absence of 100 µg ml^−1^ peptide R1. As shown in Figure 7, invasion by the parental 3D7 was efficiently (∼95%) blocked by R1, as expected. In contrast, all three of the PfAMA1/DIII-HA-expressing lines exhibited substantially higher levels of invasion, indicating partial functional complementation by the transgene. Importantly, the degree of complementation in each case (∼20%) was similar, indicating that the presence of the cleavage site mutations has no appreciable impact on PfAMA1 function at invasion. As a positive control in these assays we also included a 3D7-derived transgenic clone called 3D7-sgPfA1/HA.F5 [63] in which the endogenous 3D7-type *pfama1* gene has been entirely replaced by targeted integration of a full-length copy of the same synthetic FVO gene used in the PfAMA1/DIII-HA-expressing lines; clone 3D7-sgPfA1/HA.F5 therefore expresses only the FVO form of PfAMA1, but is otherwise isogenic with 3D7. As expected, 3D7-sgPfA1/HA.F5 was completely unaffected by the R1 peptide (data not shown), unambiguously demonstrating that the effect of R1 on invasion is solely a result of its capacity to bind PfAMA1.

### Parasites expressing shedding-resistant forms of PfAMA1 exhibit enhanced susceptibility to invasion-inhibitory anti-PfAMA1 antibodies

**Figure 8:**
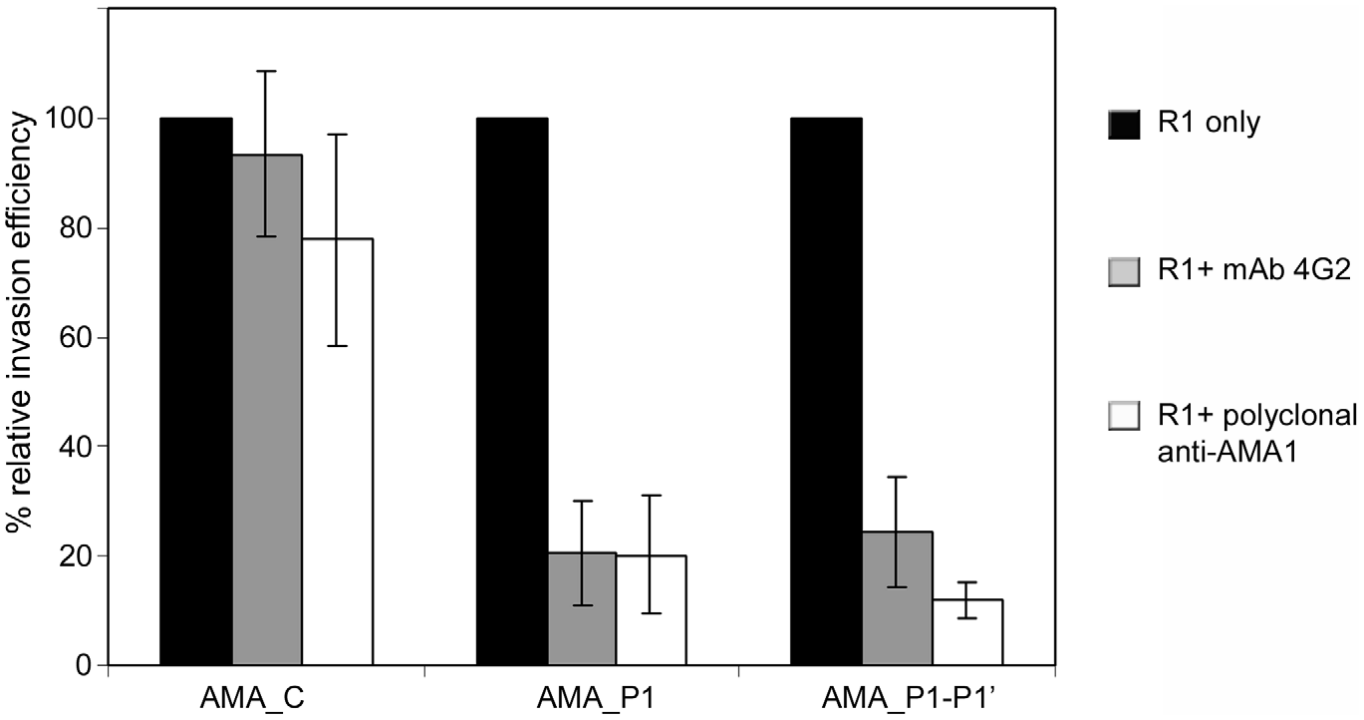
Mutations that prevent PfSUB2-mediated shedding of PfAMA1 increase sensitivity to invasion-inhibitory anti-PfAMA1 antibodies. Histogram showing the effects of anti-PfAMA1 antibodies on invasion by transgenic *P. falciparum* lines harbouring PfAMA1/DIII-HA expression constructs AMA_C, AMA_P1 or AMA_P1-P1′. All assays were performed in the presence of peptide R1 (100 µg ml^−1^ final concentration) to block the function of endogenous PfAMA1. Antibodies were used at 0.2 mg ml^−1^. Normalised relative invasion efficiencies (expressed as %) were calculated by dividing the parasitaemia values of the antibody-treated cultures with those of parallel cultures allowed to undergo invasion in the absence of antibodies (peptide R1 only). Error bars indicate standard deviation values.

Inhibition of erythrocyte invasion by antibodies against AMA1 is widely documented, and has been an important contributory factor to the continued interest in the molecule as a malaria vaccine candidate. Although the mechanistic basis of antibody-mediated invasion-inhibition remains controversial, it has been suggested that shedding of AMA1 and other parasite surface proteins may facilitate host cell entry by sequestering antibodies in the microenvironment of the invading parasite, and/or by preventing the accumulation of immune complexes on the parasite surface that might interfere with invasion [55], [57]. Having produced parasite lines expressing shedding-resistant forms of PfAMA1, we decided to take advantage of them to test this hypothesis. Lines harbouring episomal constructs AMA_C, AMA_P1 and AMA_P1-P1′ were examined for their susceptibility to two invasion-inhibitory anti-PfAMA1 antibody preparations: the rat mAb 4G2 [32], [63], [73]; and rabbit polyclonal antibodies raised against correctly folded recombinant PfAMA1 [64] (see legend to Figure 1). The invasion-inhibition experiments were performed using concentrations of antibodies that inhibit invasion by the parental 3D7 clone by no more than ∼20%, in order to best enable us to distinguish differences in susceptibility to the antibodies; preliminary experiments also demonstrated that the parental clone and AMA_C line were equally sensitive to the antibodies (Figure S7 in Text S1). In order to functionally inactivate the endogenous, normally-shed PfAMA1 expressed by the three parasite lines under investigation, thus focusing on the effects of the antibodies on the episomally-expressed transgenic PfAMA1, all assays comparing the three transgenic lines also included R1 peptide at a concentration (100 µg ml^−1^) that effectively blocks invasion by the parental 3F7 clone (see Figure 7). As shown in Figure 8, under these conditions mutant lines AMA_P1 and AMA_P1-P1′ were reproducibly more sensitive than the control AMA_C line to the invasion-inhibitory activity of the antibodies. Since these mutants differ from AMA_C only by the substitution of one (AMA_P1) or two (AMA_P1-P1′) amino acid residues that alter susceptibility to PfSUB2-mediated shedding, these results strongly suggest that shedding of PfAMA1 indeed enables the parasite to evade invasion-inhibitory antibodies.

## Discussion

This study had three primary aims: to establish the importance of intramembrane shedding of PfAMA1; to interrogate the primary sequence requirements for cleavage of PfAMA1 by PfSUB2; and to examine the functional consequences of modifications that block that shedding. An intriguing aspect of AMA1 is that, despite its conservation across the apicomplexan phylum, it is not shed in a conserved manner. Shedding of TgAMA1 is mediated exclusively by cleavage within the TMD by a rhomboid-like protease [8], and recent elegant work from Buguliskis and colleagues has implicated a *Toxoplasma* tachyzoite surface rhomboid called TgROM4 in this [23]. Like TgROM4, the *P. falciparum* orthologue of ROM4 is similarly expressed at the merozoite surface [21], and this protease may be responsible for the small fraction of PfAMA1 shedding that is mediated by intramembrane cleavage [8]. In conflict with this is evidence from another study using a cell-based *in vitro* assay of intramembrane cleavage activity, which suggested that another parasite rhomboid called PfROM1, but not PfROM4, has the capacity to shed PfAMA1 [22]. Whichever enzyme is responsible, prior to this study the biological relevance of this low-level rhomboid-mediated cleavage of PfAMA1 was unknown, and indeed we have previously speculated that it is artefactual [8]. Our ability here to severely ablate intramembrane shedding (in the absence of EGTA) by appropriate mutagenesis of the intra-TMD cleavage site, with no effect on parasite growth, supports that. We cannot formally rule out that very low level intramembrane shedding, below the level of detection by Western blot, is important. This possibility is highlighted by the observation that low-level production of the PfAMA1_52_ form could be detected under the very artificial conditions of merozoite release in the presence of EGTA, which enhances the products of intramembrane cleavage presumably due to its blockade of both invasion and PfSUB2 activity [8], [12]. This residual intramembrane shedding could be due to incomplete blockade of cleavage by the Ala550Tyr mutation, or even low-level phenotypic reversion of in culture of the mutant, given the single-crossover nature of its genomic modification. Collectively, however, we consider that the weight of evidence argues against an essential role for intramembrane cleavage of AMA1 in *P. falciparum*, a view in accord with the fact that the Ala550 residue at which intramembrane cleavage of PfAMA1 occurs is not conserved across predicted *Plasmodium* AMA1 TMD sequences; indeed several species lack an Ala residue or any of the known 'helix-destabilising' residues within the luminal 8–10 residues of their TMD (Figure S8 in Text S1) that are considered to be a signature of rhomboid substrates [61] (see [74] for a recent review of this subject). It might therefore be predicted that intramembrane cleavage of AMA1 is not a common feature in *Plasmodium*.

In previous work we had noted that the sequences flanking the PfSUB2 cleavage sites in MSP1 and PfAMA1 show no obvious similarity, and so PfSUB2 was tentatively likened to the secretases or 'sheddases', a group of metalloproteases responsible for shedding of ectodomains of membrane-associated proteins in metazoa. A common characteristic of this group of proteases is that they do not recognise specific sequence motifs but instead are thought able to cleave within any relatively disordered sequence at positions a certain distance from the membrane or a membrane distal globular domain. Using episomal expression in the parasite of epitope-tagged PfAMA1 transgenes, it was therefore not completely surprising to us that substitution with Ala of all six residues (P3-P3') flanking the PfAMA1 cleavage site, or mutation of the P1 Thr to Arg or Tyr, or the P2 Val to a Phe residue, or even substitution of both P1 and P1' residues with Tyr, had no detectable effect on the efficiency of shedding. The shed cleavage products from the AMA_M1, AMA_M2, AMA_M3, AMA_R1, AMA_Y1, AMA_Y1-Y1′ and AMA_F2 mutants co-migrated precisely on SDS PAGE with those of the parental AMA_C transgene product, leading us to conclude that cleavage of the mutants likely takes place at the same position relative to the TMD. The significance of the enhanced levels of the intramembrane cleavage product, PfAMA1_52_, present in the AMA_R1 culture supernatants is presently unclear, but the site-specificity of cleavage appeared unchanged. These conclusions were supported by our subsequent observation that duplication of a 21-residue sequence that lies between the canonical PfSUB2 cleavage site and the TMD resulted in the shedding of an appropriately larger form of the PfAMA1/DIII-HA ectodomain. Mass spectrometric mapping of affinity-purified shed AMA_ins protein – although failing to definitively assign the new site – confirmed cleavage downstream of the normal position. This finding strongly suggests that the position of cleavage is determined primarily by its distance from the parasite membrane, fully supporting the sheddase hypothesis. Our results are consistent with previous observations regarding the lack of sequence conservation at the PfSUB2 processing site in AMA1 across *Plasmodium* species [8], and also the observation that PTRAMP, a third PfSUB2 substrate [13], contains no sequence motifs similar to either the PfAMA1 or MSP1 cleavage sites. Presumably, the broad specificity of PfSUB2 enables it to cleave a range of membrane-bound substrates, largely irrespective of their primary sequence. However, the fact that not all merozoite surface proteins are shed by PfSUB2 [21] indicates some degree of discrimination. Further work will be required to establish the full repertoire of merozoite proteins that are substrates for the protease, as well as the higher order structural or other features that render a protein susceptible to PfSUB2. The fact that the AMA_del mutant, in which part of the juxtamembrane 'stalk' region was deleted, exhibited only poor cleavage may indicate that a reasonably long such region is a minimal requirement for targeting by PfSUB2.

The third part of our study addressed the effects of mutations at the processing site that interfere with cleavage. As previously observed for several other proteases, we found that the insertion of a single Pro residue at the P1 position, or two Pro residues flanking the scissile bond, very efficiently blocked shedding of the PfAMA1/DIII-HA transgene product. We had anticipated that, given the normally highly efficient shedding of PfAMA1 at invasion, expression of such episomal constructs in the parasite might be deleterious to parasite growth. In fact, we found that the cleavage-resistant AMA_del, AMA_P1, or AMA_P1-P1′ transgenes were expressed just as efficiently as the other transgene mutants, and that the lines carrying them showed no detectable growth defect. Focusing on the AMA_P1, or AMA_P1-P1′ mutants, we used antibodies to the ectodomain-located HA epitope tag to show that, consistent with the virtually complete absence of shed ectodomain released into culture supernatants, the mutant gene products were efficiently carried into host erythrocytes on the surface of invading merozoites. This proves unambiguously that complete shedding is not a prerequisite for invasion. Furthermore, using the R1 peptide, which blocks the invasion-related function of 3D7 PfAMA1 in an allele-specific manner, we showed that the FVO-based cleavage-resistant mutants complemented the function of R1-inactivated endogenous PfAMA1 as efficiently as the AMA_C-derived transgene product, convincingly demonstrating that PfSUB2-mediated shedding is not required for the function of PfAMA1 at invasion.

Given these findings, what is the explanation for our inability to introduce the P1 and P1-P1' mutations into the endogenous *pfama1* gene? One interpretation of this result is that although PfSUB2-mediated cleavage of PfAMA1 is not essential for the function of PfAMA1 during invasion, some shedding is essential at some point in the asexual blood-stage life cycle. This notion is supported by a recent study in *Toxoplasma* indicating that intramembrane cleavage of TgAMA1 is required not for invasion, but to trigger parasite replication following invasion [24]. Is it possible that PfSUB2-mediated cleavage in *P. falciparum* performs an analogous role to that of TgROM4 in *Toxoplasma*? Work is underway to address this question.

Unlike many other microneme proteins (e.g. the erythrocyte binding protein EBA-175 [21]) PfAMA1 is abundantly detected on the surface of free merozoites, and indeed its discharge onto the merozoite surface has been documented to occasionally occur even prior to schizont rupture [65], [75]. This, combined with the established importance of AMA1 in invasion and its apparent requirement to form interactions with the RON proteins, may account for its relative sensitivity to antibodies compared with that of other microneme proteins that have been examined, several of which are predominantly discharged only upon contact with the host cell (e.g. [76]). We took advantage of the episomal parasite lines expressing shedding-resistant PfAMA1/DIII-HA to examine their susceptibility to invasion-inhibition by anti-PfAMA1 antibodies. Surprisingly, we found that the transgenic parasites expressing the AMA_P1 or AMA_P1-P1′ mutants were significantly more sensitive to antibody-mediated invasion inhibition that the control AMA_C line, indicating that shedding decreases sensitivity to these antibodies. These assays focused on the transgene products only, because they were all performed in the presence of concentrations of the R1 peptide that effectively block the function of the endogenous 3D7 PfAMA1. We can only speculate on the mechanistic basis of this enhanced sensitivity. Previous knockdown studies in *Toxoplasma* using a tetracycline-regulated conditional system have indicated that TgAMA1 is normally expressed at levels well in excess of those necessary to sustain its role at invasion, since reduction of endogenous protein levels by ∼90% (upon replacement of the endogenous gene with a regulated copy) had no effect on invasion efficiency [41]. We hypothesise that if this is also the case in *Plasmodium*, and if only those PfAMA1 molecules involved in interactions with the RON complex play a productive role in invasion, it may be important for the parasite to rapidly discard excess surface PfAMA1 molecules in the presence of anti-PfAMA1 antibodies in order to prevent accumulation of antibody complexes on the parasite surface that interfere with the parasite's normally rapid passage into the nascent parasitophorous vacuole. Thus, even though our results clearly demonstrate that complete shedding from the merozoite surface is not mechanistically essential for invasion to go to completion, it may be evolutionarily advantageous for shedding of PfAMA1 to occur in an efficient manner in order to cope with the threat of host-derived anti-PfAMA1 antibodies.

In conclusion, our results suggest that shedding of PfAMA1 by PfSUB2 may perform dual functions, one of which is essential and the other of which enables the parasite to more efficiently evade the host humoral response. Shedding of apicomplexan zoite surface proteins is a widespread phenomenon [55], and our observations have obvious implications for shedding of other immunogenic parasite proteins too. Collectively, our findings imply that efficient inhibition of PfSUB2 activity by suitable drug-like inhibitors would prevent parasite growth, and – importantly – that even partial inhibition of PfSUB2 activity may enhance the efficacy of invasion-inhibitory anti-merozoite antibody responses *in vivo*.

## Materials and Methods

### Plasmid construction

Constructs AMA_M1, AMA_M2, AMA_M3, AMA_R1, AMA_Y1, AMA_Y1-Y1′, AMA_F2, AMA_P1, AMA_P1-P1', and AMA_A550Y were produced by introducing mutations by Quikchange site-directed mutagenesis (Stratagene) into plasmid pST2A-sgPfa1 [32], then (in all cases except AMA_A550Y) subcloning DNA fragments containing the mutations into plasmid pHAM-sgA1/DIII-HA [32] (here renamed AMA_C for clarity), using flanking *Avr*II and *Stu*I restriction sites. To obtain construct AMA_ins, AMA_C was digested with *Bsi*WI and *Stu*I and the excised fragment replaced by a PCR product containing an additional 65 bp encoding Glu526–Lys546 inclusive, in order to duplicate this sequence in AMA_ins. The insert was PCR-amplified from AMA_C using forward primer AMA1ins_F and reverse primer AMA1ins_R (see Table S1 in Text S1 for all primers used). Construct AMA_del was obtained by PCR-amplification of sequence encoding residues Asn519-Asp530 using primers AMA1del_F and AMA1ins_R, then subcloning the resulting PCR product back into *Spe*I/*Stu*I-digested AMA_C.

To produce integration constructs intAMA_R-TKmod and intAMA_C-TKmod, one of the multiple cloning sites was first removed from pHH-TK with restriction enzymes *Hpa*I and *Ksp*I, followed by blunting and religation, producing pHH-TKΔMCS2. The *P. berghei dhfr* 3′ UTR (PbDT3′) was then PCR-amplified from pHH1 using primers PbDT3′_ClaI_MCS_F and PbDT3′_R and subcloned into pHH-TKΔMCS2, giving rise to plasmid pHH-TKmod. Sequence including the mutation at the rhomboid cleavage site in PfAMA1 was subcloned from AMA_A550Y into intermediate plasmid PSL1180_AMA_C (see below) with restriction enzymes *Sna*BI and *Stu*I. The entire chimeric gene from this plasmid, as well as the control chimera from PSL1180_AMA_C was excised by restriction with *Xho*I and blunting the cut ends followed by digestion with *Bgl*II. This was cloned into pHH-TKmod pre-digested with *Bgl*II and *Hind*II, producing intAMA_R-TKmod and intAMA_C-TKmod.

Integration constructs intAMA_C, intAMA_P1 and intAMA_P1-P1′ were generated using AMA_C, AMA_P1 and AMA_P1-P1′ respectively. A 1071 bp 5′ targeting region of the *pfama1* gene, excluding sequence encoding the first 4 Met residues, was amplified from 3D7 genomic DNA using primers AMAint_F and AMAint_R. This was cloned into plasmids AMA_C, AMA_P1 and AMA_P1-P1′ using restriction enzymes *Hpa*I and *Avr*II, replacing the *pfama1* promoter sequence and 3′ end of the synthetic FVO *ama1* gene and resulting in a partial in-frame chimera between the targeting and synthetic coding sequences. To obtain the derivatives intAMA_C_TK, intAMA_P1_TK and intAMA_P1-P1′_TK the entire chimeric coding sequence of each construct plus its downstream *P. berghei dhfr* 3′ UTR (PbDT3′) was excised with restriction enzymes *Hpa*I and *Not*I, cloned into plasmid PSL1180 (GE Healthcare) pre-digested with *Eco*RV and *Not*I (to produce plasmids PSL1180_AMA_C, PSL1180_AMA_P1 and PSL1180_AMA_P1-P1′) then the inserts excised with restriction enzymes *Bgl*II and *Ksp*I and cloned into plasmid pHHT-TK digested with the same enzymes. All final constructs were confirmed by sequencing on both strands.

### Parasite culture and transfection

*P. falciparum* clone 3D7 was maintained and synchronized as described previously [77]. For transfection, ring-stage parasites (5–10% parasitaemia) were electroporated with 100 µg of plasmid DNA using standard procedures [11]. Episomal lines were obtained by continuous selection with 10 nM WR99210 (Jacobus Pharmaceuticals, New Jersey, USA), whereas to select for integration in parasites transfected with plasmids intAMA_P1, intAMA_C and intAMA_P1-P1′, cultures were subjected to repeated cycles of WR99210 treatment and removal as described [11]. Parasites transfected with plasmids intAMA_R-TKmod, intAMA_C-TKmod, intAMA_P1_TK, intAMA_C_TK and intAMA_P1-P1′_TK were expanded under selection with 2.5 nM WR99210 and then treated with increasing concentrations of gancyclovir (4 µM, 8 µM and 20 µM, for ∼7 days at each concentration) to select for parasites containing single copies of the TK gene resulting from single-crossover integration of the input plasmid into the *pfama1* locus.

For invasion assays, schizonts were enriched by centrifugation over cushions of 70% Percoll (GE Healthcare), added to fresh erythrocytes to obtained a parasitaemia of ∼4% and 1% haematocrit, and cultured overnight in the presence or absence of 100 µg ml^-1^ R1 peptide, with or without the addition of 0.2 mg ml^-1^ mAb 4G2 or Protein G-purified IgG from the rabbit anti-PfAMA1 antiserum Rb1, described previously [32]. Giemsa stained thin blood films were prepared 16-18 h later and parasitaemia values determined microscopically. Normalised relative invasion efficiencies were calculated by dividing the parasitaemia values of the R1-treated cultures with those of the untreated samples, or by dividing the parasitaemia values of the R1 plus antibody-treated samples with those of the R1 only-treated cultures. For purified ring preparations, schizonts enriched by centrifugation over cushions of 70% Percoll were cultured with fresh erythrocytes for 3 hours to allow rupture and reinvasion, then residual schizonts removed by repeated passage over 70% Percoll cushions as described previously [9]. For analysis of proteins released into Albumax-free culture supernatants, samples were prepared as previously described [32].

### Southern blot and diagnostic PCR

Genomic DNA from wild type and integration lines was extracted using a QIAamp DNA blood mini kit (QIAGEN), digested to completion with *Xba*I (for parasites transfected with intAMA_P1, intAMA_C, intAMA_P1-P1′, intAMA_C_TK, intAMA_P1_TK and intAMA_P1-P1′_TK) or *Nde*I (for lines transfected with intAMA_R-TKmod and intAMA_C-TKmod), electrophoresed on a 0.7% agarose gel, transferred to Hybond N+ nylon membrane (GE Healthcare) using standard procedures, and probed with [^32^P]-labelled DNA fragments, produced using the Prime-It II random primer labelling kit (Stratagene). The 3′ region of the endogenous *pfama1* gene (amplified from genomic DNA with primers AMA1Sprobe_F and AMA1Sprobe_R, Table S1 in Text S1) was used as a probe in Southern blot hybridisations. Primers used for diagnostic PCR reactions, called verAMAwt_R, verAMAint_F, and verAMAint_R are also shown in Table S1 in Text S1. The latter two are schematically represented in Figure 6A. PCR reactions were performed using high fidelity Taq polymerase (Invitrogen) according to the manufacturers' instructions, with 30 amplification cycles using an annealing temperature of 55°C and an extension time of 1 minute. As a negative control a mixture of genomic DNA from 3D7 parasites and plasmid intAMA_C was used as a template.

### Antibodies, Western blot, and indirect immunofluorescence assay (IFA)

Rabbit antibodies raised to correctly-folded recombinant FVO PfAMA1, and the polyclonal mouse anti-PfAMA1 antibody R5 have been described previously [32], [64], as has a polyclonal antibody against EBA-175 [21], mAb 4G2 [63], the anti-SERA5 mAb 24C6.1F1 [78] and the anti-MSP1 mAb X509 [79]. A polyclonal antibody against the parasitophorous vacuole protein Exp1 was a kind gift of Klaus Lingelbach, University of Marburg, Germany. For Western blot analysis, culture supernatants and pellets of synchronous highly mature schizonts were solubilised in SDS sample buffer and subjected to SDS-PAGE under reducing conditions, followed by transfer to Hybond-C extra nitrocellulose membrane (GE Healthcare). Membranes were probed with mAbs or polyclonal antibodies as described previously [32]. For IFA, air dried thin blood films were fixed in 4% (w/v) formaldehyde (Agar Scientific Ltd.) for 30 min, permeabilized for 10 min with 0.1% (w/v) Triton X100, then blocked for 1 h with 3% (w/v) bovine serum albumin. After incubation for 30 min with the HA-specific mAb 3F10 (Roche) diluted 1:200 and/or a rabbit polyclonal antiserum raised against recombinant region VI of EBA175 [21] diluted 1:500, films were washed for 5 min in PBS. Thin films were then incubated with a biotinylated goat anti-rat IgG (Chemicon) diluted 1:500, followed by incubation with FITC streptavidin (Vector Laboratories) diluted 1:500 and with Alexa Fluor 594 conjugated anti-rabbit antibody (Invitrogen) diluted 1:500. Nuclei were stained by 5 min immersion in 2 µg ml^−1^ DAPI in PBS, then washed in PBS. Samples were mounted in Citifluor (Citifluor Ltd., Canterbury, U.K.), and images collected using AxioVision 3.1 software on an Axioplan 2 Imaging system (Zeiss) using a Plan-APOCHROMAT 1006/1.4 oil immersion objective.

### Affinity chromatography and mass spectrometry

PfAMA1 fragments shed into culture supernatants were affinity-purified on a column of immobilised mAb 4G2 as previously described [12], [66]. Following SDS PAGE and staining with InstantBlue (Generon), bands corresponding to proteins of interest were excised and the proteins analysed by LC/MS/MS as previously described [80]. LC/MS/MS data were searched against the UniProt KB (release 15.5) protein database using the Mascot search engine programme (Matrix Science, UK), as well as against the predicted primary sequence of the AMA_ins transgene.

## Supporting Information

Text S1 Supplemental Figures S1 to S8, and Table S1.
(PDF)
