# Correction: Juxtamembrane Shedding of *Plasmodium falciparum* AMA1 Is Sequence Independent and Essential, and Helps Evade Invasion-Inhibitory Antibodies

**DOI:** 10.1371/annotation/72b02b2e-460a-44f4-bc87-067cfe76bfdb

**Published:** 2012-02-29

**Authors:** Anna Olivieri, Christine R. Collins, Fiona Hackett, Chrislaine Withers-Martinez, Joshua Marshall, Helen R. Flynn, J. Mark Skehel, Michael J. Blackman

The affiliation for the eighth author was incorrect. Michael J. Blackman is not affiliated with #2 but with #1 Division of Parasitology, MRC National Institute for Medical Research, Mill Hill, London, United Kingdom.

