# Supplementary material for: Juxtamembrane Shedding of Plasmodium falciparum AMA1 Is Sequence Independent and Essential, and Helps Evade Invasion-Inhibitory Antibodies
Source: PLoS Pathog. 2011 Dec 15;7(12):e1002448. doi: 10.1371/journal.ppat.1002448 (PMC3240622; doi:10.1371/journal.ppat.1002448)
Supplement: Text S1 — Supplemental Figures S1 to S8, and Table S1. (PDF) [file ppat.1002448.s001.pdf]

**Supporting information**

# Juxtamembrane Shedding of *Plasmodium falciparum* AMA1 is Sequence Independent and Essential, and helps Evade Invasion-inhibitory Antibodies

Anna Olivieri[1§], Christine R. Collins[1§], Fiona Hackett[1], Chrislaine Withers-Martinez[1], Joshua Marshall[1], Helen R. Flynn[2], J. Mark Skehel[2] and Michael J. Blackman[2*]

[1]Division of Parasitology, MRC National Institute for Medical Research, Mill Hill, London NW7 1AA, United Kingdom, and [2]Protein Analysis and Proteomics Laboratory, Clare Hall Laboratories, Cancer Research UK London Research Institute, Blanche Lane, South Mimms, Hertfordshire EN6 3LD, United Kingdom.

* E-mail: mblackm@nimr.mrc.ac.uk

[§] These authors contributed equally to this work.

**Figure S1. Southern blot confirms integration of intAMA_R-TKmod and intAMA_C-TKmod into the *pfama1* locus.** (A) Schematic of constructs designed to modify the *pfama1* locus by single cross-over homologous recombination. Sequence derived from the recodonised synthetic FVO *pfama1* gene [1-3] (light blue) is fused in frame to targeting sequence identical to that in 3D7 (light pink), to promote cross-over upstream of mutations (not shown) introduced into the TMD-encoding part of the FVO sequence in intAMA_R-TKmod. Construct intAMA_C-TKmod is identical to intAMA_R-TKmod aside from the mutations, so is designed to simply reconstitute the PfAMA1 wild-type TMD sequence. Wild-type (wt) and modified (int) *pfama1* loci are illustrated and the *Nde*I digestion patterns predicted for Southern blot analysis with the probe indicated (P) are shown. (B) Southern blot analysis of *Nde*I-digested genomic DNA extracted from parental 3D7 and transgenic 3D7_AMA_C and 3D7_AMA_R parasite lines. Signals from hybridisation of the probe with fragments derived from the modified (2.2 kDa) and unmodified (3.7 kDa) loci are indicated. Three clones (3D7_AMA_C_E9, B6 and D7 and 3D7_AMA_R_D4, F7, F9) were analysed from each transgenic line.

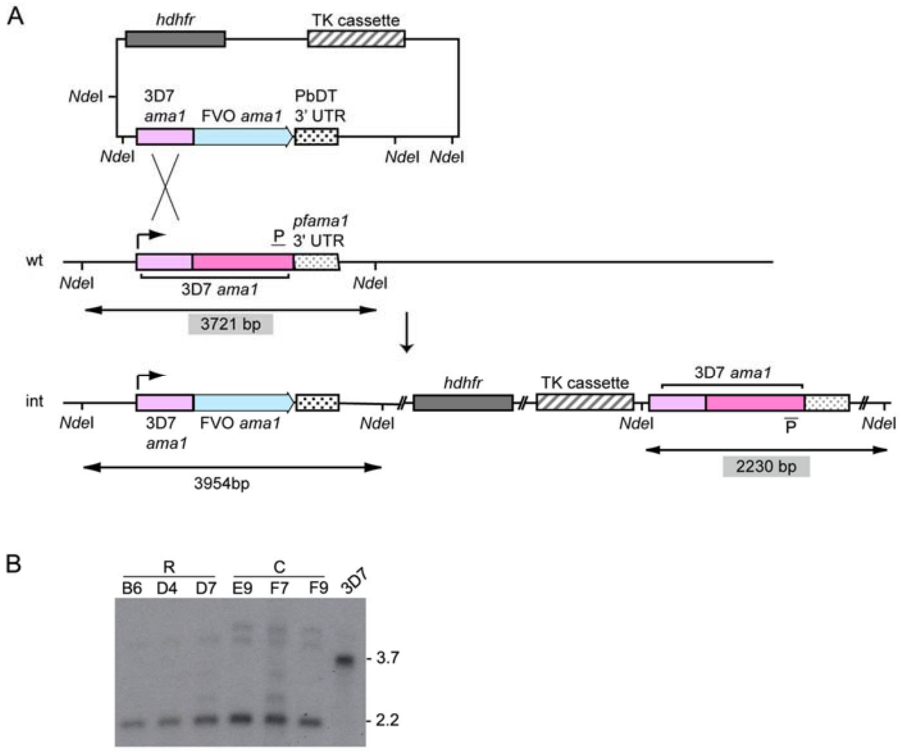

**Figure S2. Efficient inhibition of intramembrane shedding of PfAMA1 in the Ala550Tyr mutant.** Western blot titration of culture supernatants from the 3D7_AMA_C_E9 (control) and 3D7_AMA_R_D4 (Ala550Tyr) transgenic parasite lines, probed with a rabbit polyclonal antibody against PfAMA1. This detects all the products of PfAMA1 shedding, including the $PfAMA1_{52}$ species (arrowed) derived from intramembrane cleavage. By comparing the intensity of the $PfAMA1_{52}$ band in different loadings of the 3D7_AMA_C_E9 control supernatant with its intensity in a known loading of 3D7_AMA_R_D4 Ala550Tyr supernatant, it was calculated that the Ala550Tyr mutation reduces intramembrane shedding by at least 8-fold compared to the control. Loading control blots (not shown, but see Figure 1 in the main manuscript) showed that equal volumes of the undiluted culture supernatants contained equal amounts of a shed fragment of MSP1.

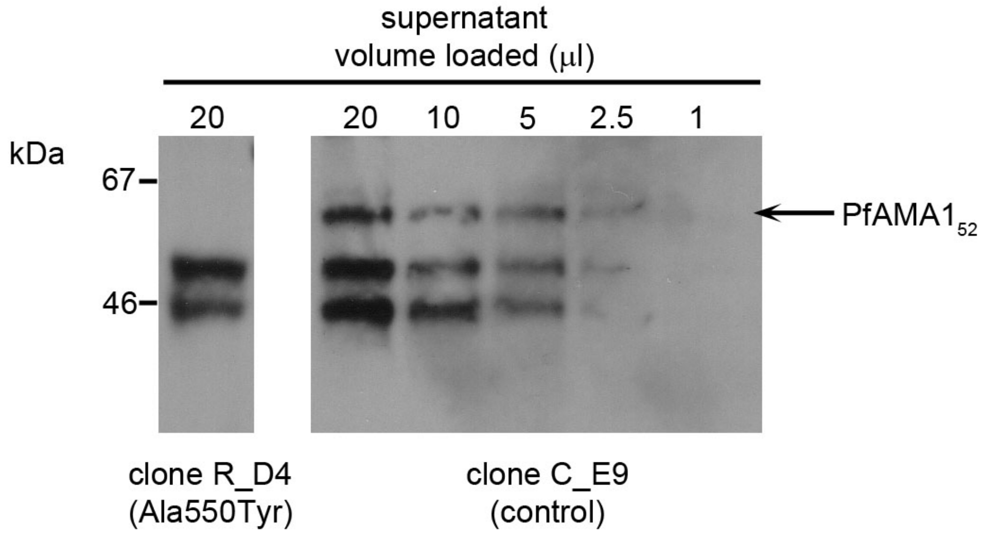

**Figure S3. Affinity purification of shed AMA_ins.** Culture supernatants collected following rupture of schizonts of the AMA_ins transgenic *P. falciparum* line were chromatographed on a mAb 4G2 column as described previously [4,5]. Eluted proteins were subjected to SDS PAGE under reducing conditions. Staining with InstantBlue (left-hand side) showed the characteristic pattern of $PfAMA1_{44}$, $PfAMA1_{48}$ and $PfAMA1_{52}$ fragments derived from shedding of endogenous PfAMA1, as well as an additional band migrating just above the $PfAMA1_{52}$ band. Western blotting showed that whilst all species were reactive with polyclonal anti-PfAMA1 serum R5, only the additional band reacted with anti-HA mAb 3F10, identifying it as the product of AMA_ins cleavage. Note that the abundant contaminating protein detected by InstantBlue staining is bovine serum albumin derived from the parasite culture supernatants, as previously observed when using this affinity-purification protocol [5].

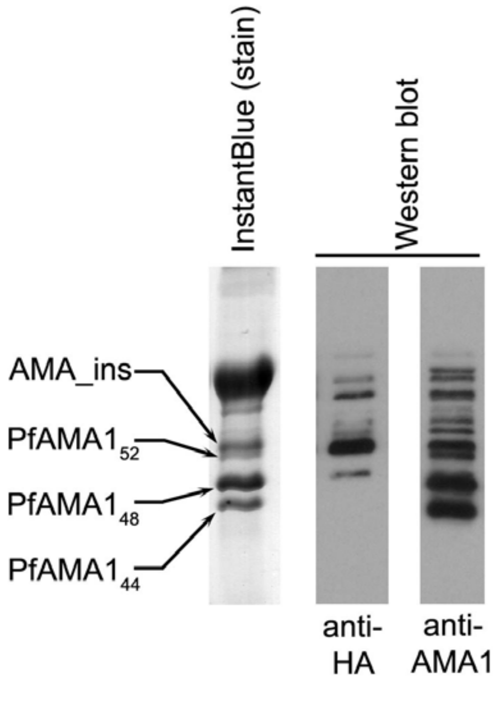

**Figure S4. Tryptic peptides identified by LC/MS/MS analysis of the shed AMA_ins polypeptide (see Figure S3).** Shown is the predicted primary sequence of the entire AMA_ins transgene (643 amino acid residues). The single HA epitope tag incorporated into domain III is shown in lower case and underlined, whilst the transmembrane domain is shaded in grey. Positions of prosequence cleavage (on the N-terminal side of Ile97 [4]) and the predicted new PfSUB2 cleavage site in this construct, are arrowed. Sequence spanned by peptides identified in LC/MS/MS analysis of in-gel tryptic digests of the shed AMA_ins polypeptide are highlighted in yellow.

```
MRKLYCVLLLSAFEFTYMINFGRGQNYWEHPYQKSDVYHPINEHREHPKEYEYPLHQEHTYQQEDSGED
ENTLQHAYPIDHEGAEPAPQEQNLFSSIEIVERSNYMGNPWTEYMAKYDIEEVHGSGIRVDLGEDAEVA
GTQYRLPSGKCPVFGKGIIIENSKTTFLKPVATGNQDLKDGGFAFPPTNPLISPMTLNGMRDFYKNNEY
VKNLDELTLCSRHAGNMNPDNDKNSNYKYPAVYDYNDKKCHILYIAAQENNGPRYCNKDQSKRNSMFCF
RPAKDKLFENYVYLSKNVVDNWEEVCPRKNLENAKFGLWVDGNCEDIPHVNEFSANDLFECNKLVFELS
ASDQPKQYEQHLTDYEKIKEGFKNKNADMIKSAFLPTGAFKADRYKSHGKGYNWGNYNRETQKCEIFNV
KPTCLINDKSYIATTALSHPIEVEHNFPCSLYKDEIKKEIERESypydvpdyaDEGNKKIIAPRIFISD
DKDSLKCPCDPEMVSQSTCRFFVCKCVERRAEVTSNNEVVVKEEYKDEYADIPEHKPTYDNMKEEYKDE
YADIPEHKPTYDNMKIIIASSAAVAVLATILMVYLYKRKGNAEKYDKMDQPQHYGKSTSRNDEMLDPEA
SFWGEEKRASHTTPVLMEKPYY
```

**Figure S5. Quantitation of PfAMA1/DIII-HA transgene expression relative to levels of endogenous PfAMA1 in transfected parasites.** Western blot of schizont extracts (top) and culture supernatants (bottom) from *P. falciparum* 3D7 lines harbouring expression constructs AMA_ins or AMA_C (see Figure 1 of the main paper for details of the transgenic proteins), probed in all cases with a rabbit polyclonal antibody against PfAMA1. This detects the products of both the endogenous gene and the transgene, which in the case of AMA_ins (asterisked) migrates on SDS PAGE as a higher molecular weight species than the endogenous product. By comparing the intensity of the AMA_ins band with that of the endogenous equivalent in tracks loaded with different amounts of extract, it was calculated that ~15% of the total PfAMA1 was derived from the episomal transgene.

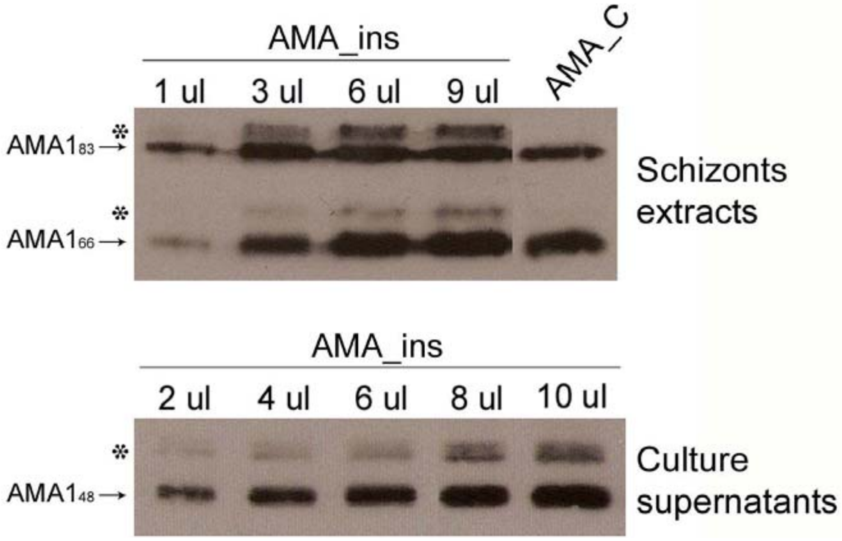

**Figure S6. Partial co-localisation of transgenic shedding-resistant PfAMA1/DIII-HA and $MSP1_{19}$ in newly-invaded rings.** IFA of newly-invaded rings of the parasite line harbouring construct AMA_P1-P1´, probed with anti-HA mAb 3F10 (green) to detect the AMA_P1-P1´ transgene product, and a mAb specific for $MSP1_{19}$ (red). DAPI was used to visualise parasite DNA (blue).

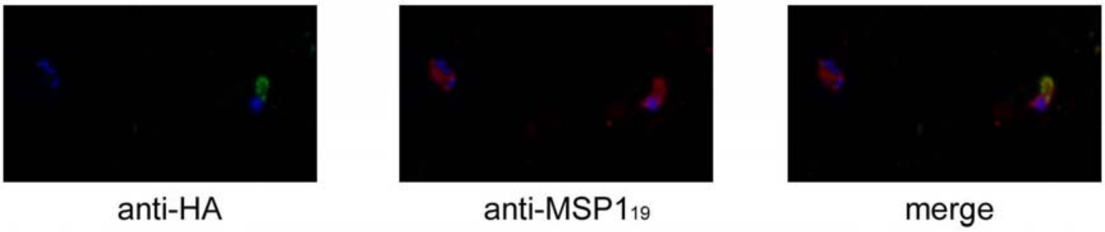

**Figure S7. Determination of sub-inhibitory concentrations of anti-PfAMA1 mAb 4G2.** Invasion inhibition experiments to determine concentrations of purified mAb 4G2 that inhibit invasion of the parental 3D7 *P. falciparum* clone by no more than ~20% of no-antibody controls. Shown are the results of triplicate experiments. Error bars represent standard deviation values. Similar results were obtained with purified IgG from the rabbit polyclonal anti-PfAMA1 serum.

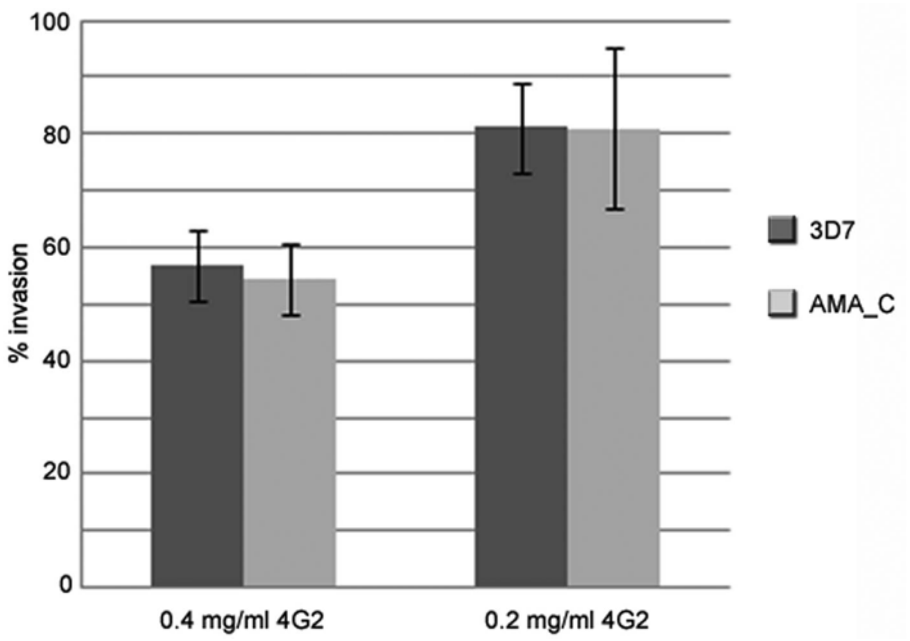

**Figure S8. Multiple alignment of the *Plasmodium* and *T. gondii* AMA1 TMD.** Shown is a multiple alignment of partial predicted sequences of AMA1 from *P. falciparum* (Pf), *P. reichenowi* (Pr), *P. vivax* (Pv), *P. knowlesi* (Pk), *P. fragile* (Pfrag), *P. chabaudi* (Pc), *P. berghei* (Pb), *P. yoelii* (Py) and *T. gondii* (Tg). The predicted TMD is shaded. The established sites of intramembrane cleavage in the Pf and Tg sequences are indicated by arrows. Helix-destabilising residues Gly, Ser, Pro and Gln, thought to be important for rhomboid cleavage [6,7] are entirely absent from the luminal regions of the Pc, Pb and Py TMD sequences. The last amino acid residue of each partial sequence shown is indicated by a number.

```
Pf    STCNFFVCKCVERRAEVTSNNEVVVKEEYKDEYADIPEHKPTYDKMKIIIASSAAVAVLATILMVYLYKRKGNAEKYDKMDE      581
Pr    STCHFFVCKCVERRTEVTSNNEVVVKEEYKDEYADIPDHKPAYDKMKIIIASSAAIAILATILMVYLYKRKTNAEKYDKMDQ      573
Pv    STCNFYVCNCVEKRAEIKENNQVVIKEEFRDYY-ENGEEKSNKQMLLIIIGITGGVCVVALASMAYFRKKANNDKYDKMDQA      522
Pk    STCNFYVCNCVEKRAEIKENNEVIIKEEFKEDY-ENPDGKHKKKMLLIIIGVTGAVCVVAVASLFYFRKKAQDDKYDKMDQA      522
Pfrag STCNFYVCNCVEKRAEIKENNEVAIKEEFKQDY-QYAQGESKNQMLLIIIGITGGVCVVALASMFYFRKKAHNDKYDKMEQA      522
Pc    SSCNFFVCNCVEKRQFISENNEVEIKDEFKSEY-----ESPINQRMLIIIIILIATGAILASLLIFYFFKSNKPGDDYDKMGQ      517
Pb    SSCNFYVCNCVEKRQYIAENNDVEIKEELEVHM-----KAHQTREVIVIIIFICVGIILVILLVGYFFKSNKKGENYDRMGQ      515
Py    STCDFYVCSCVEQRQYIAENNDVIIKEEFIGDY-----ENPNQKLLVIIILIGIGIIIVILLVAYYFKSGKKGENYDRMGQA      517
Tg    GACDVQACKRQKTSCVGGQIQST-----SVDCTADEQNECGSNTALIAGLAVGGVLLLALLGGGCYFAKRLDRNKGVQAAHH      493
```

**Table S1. Oligonucleotide primers used in this study.**

| Primer name | Sequence (5'-3') | Incorporated restriction enzyme site |
|---|---|---|
| AMA1ins_F | CAGCGTACGATAACATGAAGGAAGAATACAAGGATGAATACG | *Bsi*WI |
| AMA1ins_R | GACAGGCCTCTGGATCCAACATCTCATCGTTC | *Stu*I |
| AMA1del_F | CAGACTAGTAACAACGAAGTTGTCGTGAAGGAAGAATACAAGG ATATGAAGATCATCATCGCTAGTTCTGCTG | *Spe*I |
| AMAint_F | ACTGGTTAACGAAGAAGTTCATGGTTCAGG | *Hpa*I |
| AMAint_R | ACTGCCTAGGAGCTATAATTTTTTTATTCCCTTCATC | *Avr*II |
| AMA1Sprobe_F | CAGATGATAAAGACAGTTTAAAATGCC | |
| AMA1Sprobe_R | AGAGAAGCTGATTATATCAGACGTTG | |
| verAMAint_F | AATCCATGGACGGAATATATGGC | |
| verAMAint_R | CCGATGACAAGGATTCCCTCAA | |
| verAMAwt_R | GCCCATGTGACCCTGAAATGGT | |
| IIIA/IIIY_F | CGATAACATGAAGATCATCATCTATAGTTCTGCTGCTGTCGC | |
| IIIA/IIIY_R | GCGACAGCAGCAGAACTATAGATGATGATCTTCATGTTATCG | |
| PbDT3'_ClaI_MCS_F | GGCCCCTTTATCGATCCGCGGGGAGGACTAGTCCGAAGATCTT TGTTAACCGGATATGGCAGCTTAATGTTCG | *Cla*I, *Sac*II, *Spe*I, *Bgl*II, *Hpa*I, *Hinc*II |
| PbDT3'_R | CTCGCGGCCGCGGCGCCCTACCCTGAAGAAGAAAAGTCCGAT GATGTTGTGGAC | *Nar*I |

## References

1. Pizarro JC, Normand BV, Chesne-Seck ML, Collins CR, Withers-Martinez C, et al. (2005) Crystal structure of the malaria vaccine candidate apical membrane antigen 1. Science 308: 408-411.

2. Collins CR, Withers-Martinez C, Bentley GA, Batchelor AH, Thomas AW, et al. (2007) Fine Mapping of an Epitope Recognized by an Invasion-inhibitory Monoclonal Antibody on the Malaria Vaccine Candidate Apical Membrane Antigen 1. J Biol Chem 282: 7431-7441.

3. Collins CR, Withers-Martinez C, Hackett F, Blackman MJ (2009) An inhibitory antibody blocks interactions between components of the malarial invasion machinery. PLoS Pathog 5: e1000273.

4. Howell SA, Withers-Martinez C, Kocken CH, Thomas AW, Blackman MJ (2001) Proteolytic processing and primary structure of Plasmodium falciparum apical membrane antigen-1. J Biol Chem 276: 31311-31320.

5. Howell SA, Well I, Fleck SL, Kettleborough C, Collins CR, et al. (2003) A single malaria merozoite serine protease mediates shedding of multiple surface proteins by juxtamembrane cleavage. J Biol Chem 278: 23890-23898.

6. Urban S (2010) Taking the plunge: integrating structural, enzymatic and computational insights into a unified model for membrane-immersed rhomboid proteolysis. Biochem J 425: 501-512.

7. Strisovsky K, Sharpe HJ, Freeman M (2009) Sequence-specific intramembrane proteolysis: identification of a recognition motif in rhomboid substrates. Mol Cell 36: 1048-1059.
